# Inhibitory receptors of plasmacytoid dendritic cells as possible targets for checkpoint blockade in cancer

**DOI:** 10.3389/fimmu.2024.1360291

**Published:** 2024-03-05

**Authors:** Laura Tiberio, Mattia Laffranchi, Giovanni Zucchi, Valentina Salvi, Tiziana Schioppa, Silvano Sozzani, Annalisa Del Prete, Daniela Bosisio

**Affiliations:** ^1^ Department of Molecular and Translational Medicine, University of Brescia, Brescia, Italy; ^2^ Department of Molecular Medicine, Laboratory Affiliated to Institute Pasteur-Italia, Sapienza University of Rome, Rome, Italy; ^3^ IRCCS Humanitas Research Hospital, Milan, Italy; ^4^ IRCCS Neuromed, Pozzilli, IS, Italy

**Keywords:** BDCA-2, ILT7, pDC exhaustion, C-type lectin receptor, TLR7, checkpoint inhibitors, interferon alpha

## Abstract

Plasmacytoid dendritic cells (pDCs) are the major producers of type I interferons (IFNs), which are essential to mount antiviral and antitumoral immune responses. To avoid exaggerated levels of type I IFNs, which pave the way to immune dysregulation and autoimmunity, pDC activation is strictly regulated by a variety of inhibitory receptors (IRs). In tumors, pDCs display an exhausted phenotype and correlate with an unfavorable prognosis, which largely depends on the accumulation of immunosuppressive cytokines and oncometabolites. This review explores the hypothesis that tumor microenvironment may reduce the release of type I IFNs also by a more pDC-specific mechanism, namely the engagement of IRs. Literature shows that many cancer types express *de novo*, or overexpress, IR ligands (such as BST2, PCNA, CAECAM-1 and modified surface carbohydrates) which often represent a strong predictor of poor outcome and metastasis. In line with this, tumor cells expressing ligands engaging IRs such as BDCA-2, ILT7, TIM3 and CD44 block pDC activation, while this blocking is prevented when IR engagement or signaling is inhibited. Based on this evidence, we propose that the regulation of IFN secretion by IRs may be regarded as an “innate checkpoint”, reminiscent of the function of “classical” adaptive immune checkpoints, like PD1 expressed in CD8+ T cells, which restrain autoimmunity and immunopathology but favor chronic infections and tumors. However, we also point out that further work is needed to fully unravel the biology of tumor-associated pDCs, the neat contribution of pDC exhaustion in tumor growth following the engagement of IRs, especially those expressed also by other leukocytes, and their therapeutic potential as targets of combined immune checkpoint blockade in cancer immunotherapy.

## 1 Introduction

The concept of “immune checkpoint” is currently extending to proteins others than CTLA-4 and PD1, provided their capability to limit immune responses to a physiologic range while minimizing tissue damage. As a consequence, the number of novel potential targets for checkpoint inhibition to awake the immune system against tumors is rapidly growing, together with the envisioned combinations to develop more effective and patient-tailored cancer therapies (1–3). A particular emphasis is being placed on checkpoints expressed by innate immune cells. Indeed, the combined targeting of innate and adaptive checkpoints would unleash T-cell–mediated tumor killing also by rescuing the activation of the innate arm of immunity (4, 5). Also, innate checkpoint targeting could turn strategical when genetic instability prevents the success of T-cell-targeted checkpoint blockade, given that innate activation is independent of neoantigen recognition (6). In this scenario, a thorough understanding of the biology of novel immune checkpoints is a fundamental need for the definition of innovative therapeutic strategies.

**Figure 1:**
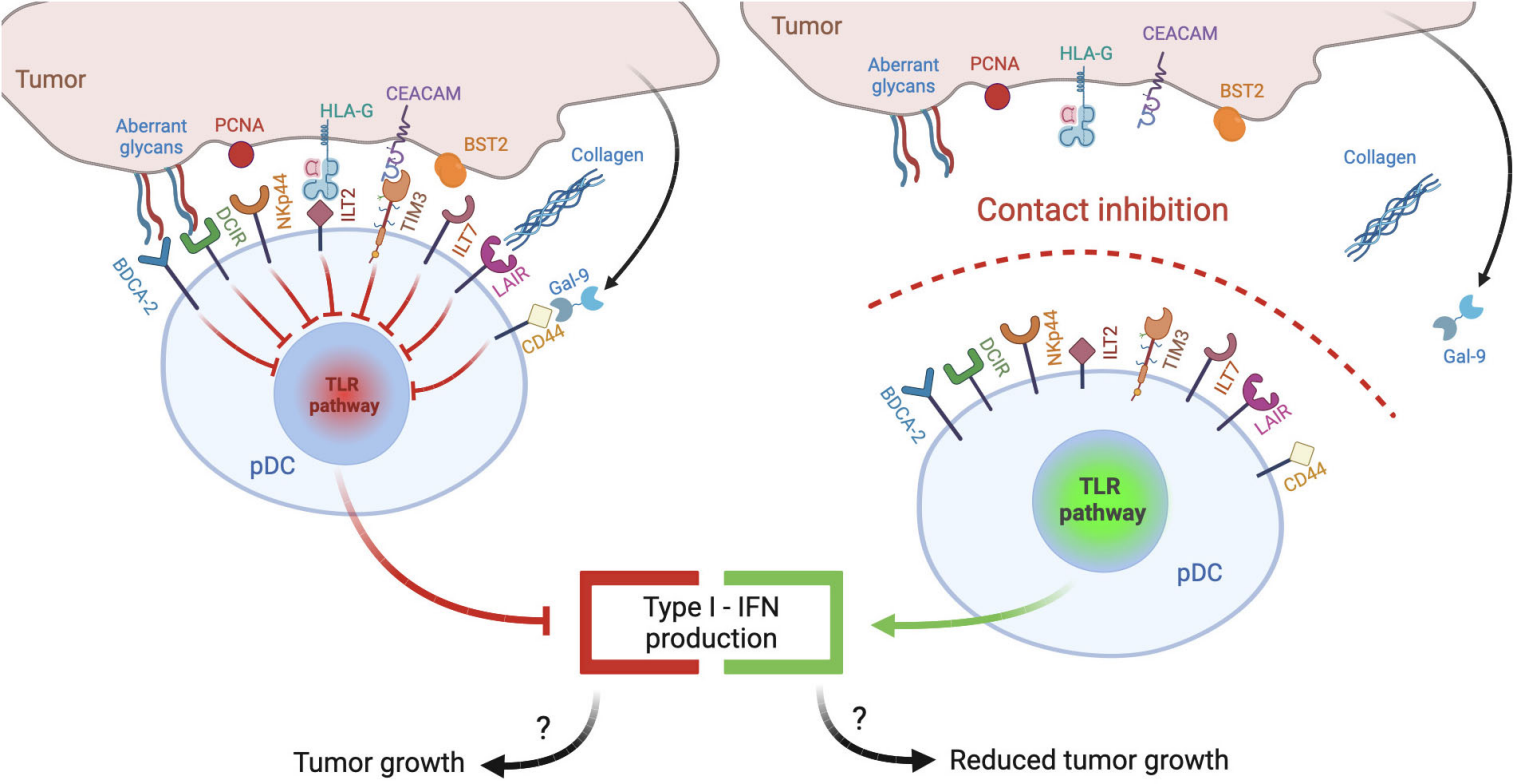
pDC inhibitory receptors as immune checkpoints and potential targets for anticancer immunotherapy. Different ligands from tumor cells may engage inhibitory receptors (IRs) expressed on pDCs and reduce pDC activation and type I IFN production, potentially leading to poor antitumor responses (left panel). Therapeutic strategies aimed at preventing IR engagement by ligand-bearing tumors may restore antitumoral pDC activity (right panel).

Plasmacytoid dendritic cells (pDCs) represent a rare subset of dendritic cells characterized by the ability to secrete massive amounts of type-I interferons (IFNs), thus eliciting antiviral and antitumor responses (7). This review explores the hypothesis that tumor microenvironment, similar to chronic viral infections, may reduce the release of type-I IFNs by engaging inhibitory receptors (IRs) expressed by pDCs (**Figure 1**, left panel): following a brief overview of pDC biology and of general mechanisms of pDC impairment in tumors, we will review the panel of IRs, collect evidence concerning their contribution in the generation of exhausted tumor-associated pDCs (TA-pDCs) and describe existing blocking strategies to rescue the anticancer potential of this cell type. In this light, we hypothesize that IRs should be regarded as “innate immune checkpoints” and further studied as potential targets for checkpoint blockade in cancer immunotherapy (**Figure 1**, right panel).

Articles referenced in the text specifically dealing with pDC IRs were searched in PubMed database from inception to December 2023 using as search terms: (“name of the receptor”[MeSH Terms] OR “name of the receptor”[All Fields] OR “alternative name/s of the receptor”[All Fields]) AND (“human plasmacytoid dendritic cells”[MeSH Terms] OR “human plasmacytoid dendritic cells”[All Fields] OR “human pDCs”[All Fields]). Retrieved papers where manually screened and were selected if related to IR characterization/biology or cancer or autoimmunity. Only inhibitory receptors of human pDCs were analyzed. Additional literature was added to draw up the more general parts of the review, concerning pDC pathophysiology and IR categorization.

## 2 Overview of pDC biology

Human pDCs, a rare population of innate cells accounting for 0.1-0.5% of mononuclear cells (8), are continuously produced in the bone marrow by both myeloid and lymphoid precursors (9). Very recently, the ontogeny of pDC has been debated and a proposal of a reclassification of their name was formulated (10, 11). Mouse data and studies on patients with combined immunodeficiencies highlighted the role of the transcription factors TCF4 (also known as E2-2), IRF8 and Ikaros family zinc finger 1 (IKZF1) for pDC differentiation (12–14). Phenotypic markers of human pDCs are blood DC antigen 2 (BDCA-2/CD303; also known as C-type lectin 4C -CLEC4C-), blood DC antigen 4 (BDCA-4/CD304; also known as C-type lectin 4A -CLEC4A-), Immunoglobulin -like transcript 7 (ILT7, also known as leukocyte immunoglobulin-like receptor subfamily A member 4 -LILRA4) and the receptor for IL7; in addition, human pDCs express other non-specific markers such as CD4, CD45RA, CD68, ILT3 and CD123 (IL-3 receptor) (15). In mice, pDCs are characterized by the expression of surface markers CD45R (B220), CD45RA, Ly-6C, Siglec-H, and BST2 (CD317/PDCA-1) (16).

Under physiologic conditions, human pDCs recirculate through lymphoid organs via peripheral blood (8). Lymph node entry occurs across high endothelial venules that express the ligands of L-selectin, CXCR4 and CMKLR1 (recently named Chemerin1, (17)) that are constitutively expressed by resting, immature pDC (18). Upon inflammatory conditions, human pDCs can enter the lymph nodes draining the target tissues guided by the acquired responsiveness to CCR7 ligands (16, 19–21). The functional role of CMKLR1- or CCR6/CCR10-mediated recruitment of human pDCs to non-lymphoid tissues has been documented during pathological conditions such as autoimmune, allergic and infectious diseases as well as in tumors (18, 22, 23). Human pDC can also migrate in response to chemotactic molecules released after tissue damage such as adenosine, formyl peptides and C3a and C5a anaphylotoxins (24–26).

pDCs were initially characterized as “natural interferon producing cells” due to their unique capability to secrete massive levels of type I IFNs (especially IFN-α) but also type III IFN (27, 28). Indeed, type I and type III IFNs account for about 60% of novel transcripts of activated pDCs (29). Moreover, pDCs also secrete proinflammatory cytokines and chemokines and were reported to present antigens to T lymphocytes (30, 31). Recent studies suggest that pDCs are a heterogeneous population, although several questions regarding pDC subsets and functional plasticity remain unanswered (15). In humans, the expression of CD2 was proposed to discriminate two different IFN-α producing pDCs subsets according to CD2 expression, with the CD2high subset being more effective in IL-12 secretion, in triggering naïve T lymphocyte proliferation and with a significant survival advantage over CD2low expressing pDC during stress conditions (32, 33). A CD5+CD81+CD2high human pDC subset, defined as Axl+ DC, was also identified. This “non-canonical” pDC subset was found unable to produce type I IFNs but endowed with the ability to stimulate B cells and promote the development of T regulatory (Treg) cells (34). However, these observations were recently challenged by a different view, supporting the idea that pDC diversification and functional specialization could occur upon activation and independently of pre-existing heterogeneity (35).

The ability to secrete huge amounts of type I IFN makes pDCs crucial in antiviral immune responses (31) against both RNA and DNA viruses (36–38). Of note, impaired secretion of type I and III IFNs caused by heterozygous null mutations in IRF7, a nonredundant transcription factor for IFN production, was associated to life-threatening H1N1 influenza A virus or SARS-CoV2 infections (39, 40). To accomplish this role, pDCs are equipped with innate immune receptors, primarily represented by elevated levels of TLR7 and TLR9. In particular, TLR7 detects ssRNA viruses, but also endogenous RNA and synthetic oligoribonucleotides or imidazoquinoline compounds. TLR9 recognizes DNA containing unmethylated CpG-rich DNA sequences, endogenous DNA and synthetic CpG DNA. The engagement of TLR7 and TLR9 activates the recruitment of the adapter protein MyD88 leading to the IRF7-mediated secretion of type I IFN and to the NF-kB-mediated secretion of proinflammatory cytokines (41). Studies using synthetic oligonucleotides demonstrated that the two pathways are spatially and temporally distinct, depending on the subcellular compartments in which these TLRs encounter their ligands (42, 43). In addition to TLRs, functional activation of cytosolic DNA-sensors including cyclic GMP-AMP (cGAMP) synthase (cGAS), stimulator of IFN gene (STING) and the dsRNA-sensor RIG-I in human pDCs has been recently described (44, 45). Virus-activated human pDCs can sustain NK cell functions by inducing NK cell migration and promoting IFN-γ secretion and NK cell cytotoxicity (46–48).

Besides their role in innate immunity, pDCs also regulate the activation of adaptive immune responses. Upon activation, pDCs increase the expression of major histocompatibility complex (MHC) and costimulatory molecules and were described to present antigens, both particulate and cell-associated, to CD4+ T cells and cross-present antigens to CD8+ T cells (49, 50). These functional pDC properties were demonstrated by specifically targeting receptors involved in antigen delivery, such as members of the C-type lectin family (CLR, such as BDCA-2, DEC-205 and DCIR, see further) or the immunoglobulin receptor FcγRII (CD32), with specific antibodies coupled to antigens, which were properly endocytosed, processed and presented (51–55). Activated pDCs secrete T-cell recruiting chemokines (18) and promote Th-polarization and differentiation (19, 56–58). Finally, type I IFNs and IL-6 released by pDCs contribute to drive memory B cell differentiation into effector plasma cell (59).

pDCs also potentially play a relevant role also in eliciting antitumor responses, which share many functional similarities with antiviral immunity (7). Indeed, type I IFNs enhance NK cell cytotoxicity against tumor cells (60, 61), modulate the activity and/or survival of lymphocytes (62, 63), suppress the generation of tumor associated macrophages (64) and also display direct antitumoral activities by inducing apoptosis and inhibiting the release of proangiogenic factors (65–68). However, the timing and duration of type-I IFN release critically condition the efficacy of antitumor responses, as recently reviewed elsewhere (69, 70), suggesting that pDC activation needs to be tightly regulated.

## 3 Impairment of pDC functions in tumors

**Figure 2:**
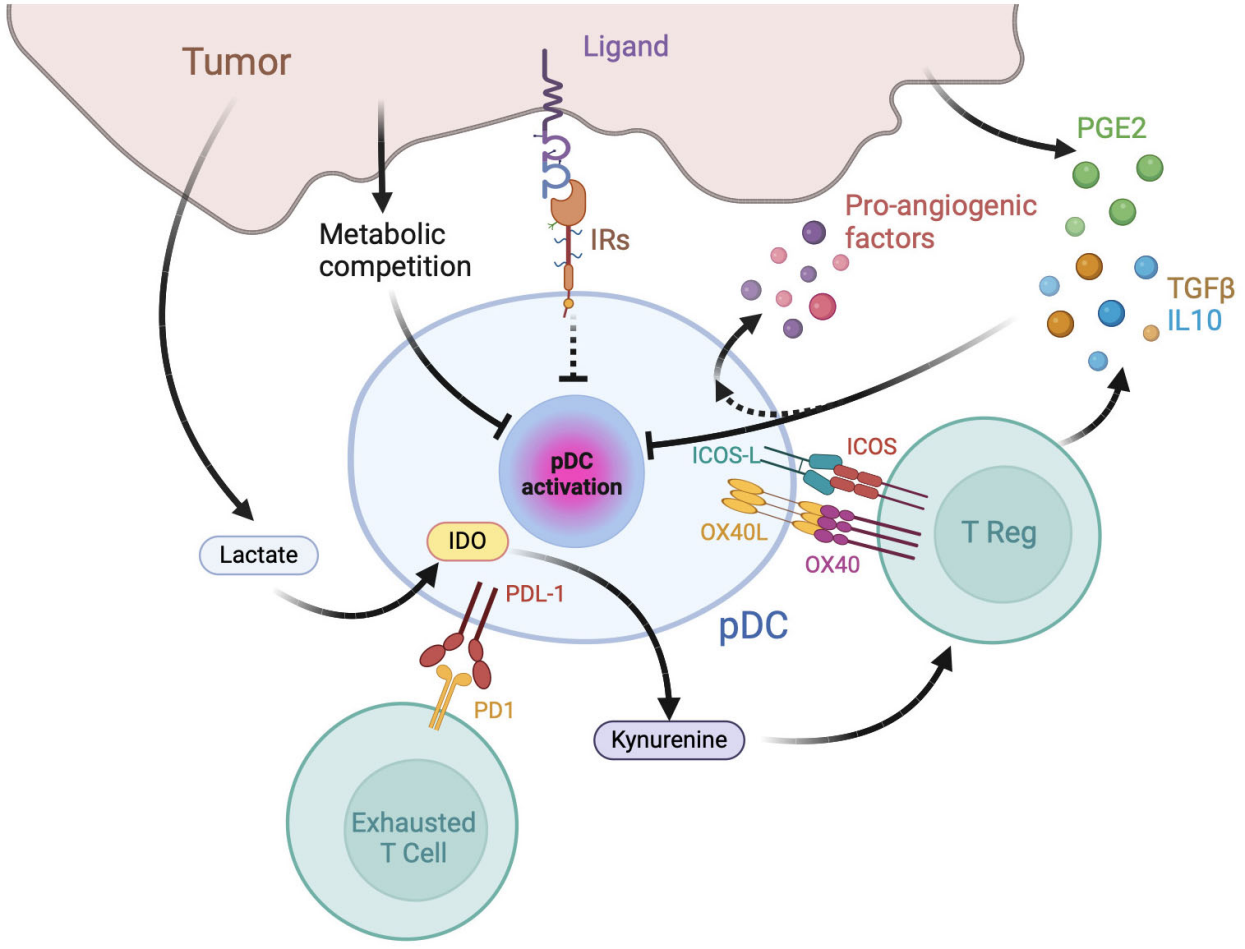
Mechanisms of pDC exhaustion and pDC-dependent immune-suppression in tumors. The tumor microenvironment is enriched in immunosuppressive cytokines, catabolites and hormones capable of inhibiting pDC maturation and type I IFN production, such as prostaglandin E2 (PGE2), TGFβ, and IL-10. pDCs favor tumor growth by inducing effector T cell exhaustion, T reg activation and by secreting pro-angiogenic factors. IR: inhibitory receptors; IDO: Indoleamine 2,3-dioxygenase.

Besides being directly associated with two major types of primary liquid neoplasia, namely Blastic pDC Neoplasm (BPDCN) and Mature pDC Proliferation (MPDCP) (71), tumor infiltration by pDCs is reported in several human solid malignancies including melanoma, head and neck cancer, ovarian carcinoma and breast cancer (72, 73). Yet, tumor-associated pDCs (TA-pDCs) generally present a dysfunctional immature phenotype, with decreased secretion of IFN-α and inability to induce appropriate T cell responses and were described as negative prognostic markers in oral, ovarian, melanoma breast cancers and others human malignancies (74–78). The following paragraphs will briefly overview the general mechanisms of pDC induction of immunosuppression and exhaustion (**Figure 2**).

### 3.1 Mechanisms of pDC-dependent immunosuppression in cancer

TA-pDCs exploit several immunosuppressive molecular mechanisms contributing to the establishment of a tolerogenic, protumor microenvironment (73). Among the best characterized, the expression of OX40L and ICOSL, two surface molecules involved in Th2/Treg activation (79–81), were described to promote an immunosuppressive milieu by secreting TGF-β and IL-10 (82–84). Indeed, Treg activation by ICOSL+ pDCs was reported in several human cancers, including melanoma (85), gastric (86), ovarian (80), glioma (87), breast (81), liver (88, 89) and thyroid gland cancers (90). Increased frequencies of OX40L+ pDC and Th2 T cells were detected in the circulation of melanoma patients (85), consistent with the Th2-skewing role OX40L expressed by TA-pDCs, as demonstrated in a melanoma mouse model (85). Moreover, circulating pDCs from multiple myeloma patients were found to express high levels of the immune checkpoint ligand PDL1 (91). On the contrary, in the presence of PDL1-blocking antibodies, pDCs promoted T cell proliferation and NK cytotoxicity in patients (91). In accordance, in non-small cell lung cancer patients undergoing anti-PDL1 therapy, a high intra-tumoral pDC signature was associated to improved survival (92). TA-pDCs were also shown to secrete immunosuppressive and tumor-promoting mediators. Indoleamine 2,3-dioxygenase expressing (IDO+) pDCs from melanoma-draining lymph nodes mediated active immunosuppression *in vitro* and caused profound local T cell anergy *in vivo* through the direct activation of Foxp3+ Tregs which, in turn, upregulated the expression of PDL1 on mouse DCs (93). TA-pDCs recovered in ascites from ovarian tumor patients secreted the proangiogenetic factors CXCL8 and TNF-α (94), while in non-small cell lung cancer patients, tumor-infiltrating pDCs were reported to cause tumor proliferation via the pro-angiogenic effects of IL-1α (95). A direct demonstration of the detrimental role of TA-pDCs comes from a glioma mouse model, where pDC depletion increased survival by reducing the number of infiltrating Tregs and their ability to secrete IL-10 (87). Similarly, in mouse models of breast cancer bone metastasis, pDC depletion resulted in an overall decreased tumor burden and bone loss via the activation of CD8+ T cells and a Th1-oriented immune response (96).

### 3.2 Mechanisms of pDC exhaustion in tumor microenvironment

The above described tolerogenic/hypo-functional state of TA-pDCs is induced by complex and often tumor-type specific molecular mechanisms (97, 98). Generally, however, the tumor microenvironment is enriched in immunosuppressive cytokines and hormones capable of inhibiting pDC maturation and type I IFN production, such as prostaglandin E2 (PGE2), TGFβ, and IL-10 (79, 82, 84, 99). These mediators are produced both by tumor cells and infiltrating immune cells, including Tregs that pDCs contribute to foster at the tumor site (83), thus establishing a feedback loop favoring tumor progression. Tumor-derived PGE2 and TGF-β were shown to act in synergy to inhibit the production of IFN-α and TNF-α induced in TLR7- and TLR9-triggered pDCs, by decreasing TLR membrane expression or by blocking TLR downstream signaling (99). This finding is consistent with the reduced capability of TA-pDCs in head and neck cancer patients to secrete type I IFNs as compared to circulating pDCs (100). The reduced expression of TLR7 and TLR9 induced by the immunosuppressive tumor microenvironment in pDCs was also demonstrated in ovarian and breast cancers (79, 81, 83, 84, 101). Conversely, PGE2-exposed pDCs release CXCL8, a chemokine that promotes tumor cell proliferation, migration/invasion and stimulates angiogenesis (73, 102). Indeed, pDCs recruited in malignant ascites from ovarian cancer patients can induce angiogenesis through the production of TNF-α and CXCL8 (94). In addition, increased serum levels of IL-10 in hepatocellular carcinoma patients were reported to induce a substantial reduction in circulating pDCs, which also displayed an immature phenotype with decreased HLA-DR, CD80, and CD86 expression (73, 103). Aberrant release of DAMPs and proinflammatory cytokines, especially TNF-α, contributes to human pDC hypo-functionality as well (94). For example, in virus-associated human cervical cancer, the production of type-I IFNs was impaired by HMGB1 secreted by transformed keratinocytes (104). Persistent stimulation of TLRs by nucleic acids released by tumor necrotic cells may also contribute to TA-pDC exhaustion like in chronic viral infections (105, 106).

Oncometabolites, such as lactate, create a microenvironment that is metabolically disadvantageous for several immune cells including pDCs (107). In mouse breast cancer, elevated lactate levels impaired the production of type I IFNs by pDCs and increased tryptophan metabolism and kynurenine, which participate in the activation of Tregs (108). In addition, in some tumor microenvironments, pDCs have to compete with tumor cells for nutrients, which are crucial for the highly metabolically demanding production of IFNs (109, 110).

All the above cited mechanisms affect different immune cells within tumors. Paragraph 4 of this review will explore the hypothesis that an additional, pDC-specific mechanism, may exist, namely the engagement of inhibitory receptors by ligand-expressing tumors.

## 4 IRs expressed by pDCs and their role in physiology and tumors

**Figure 3:**
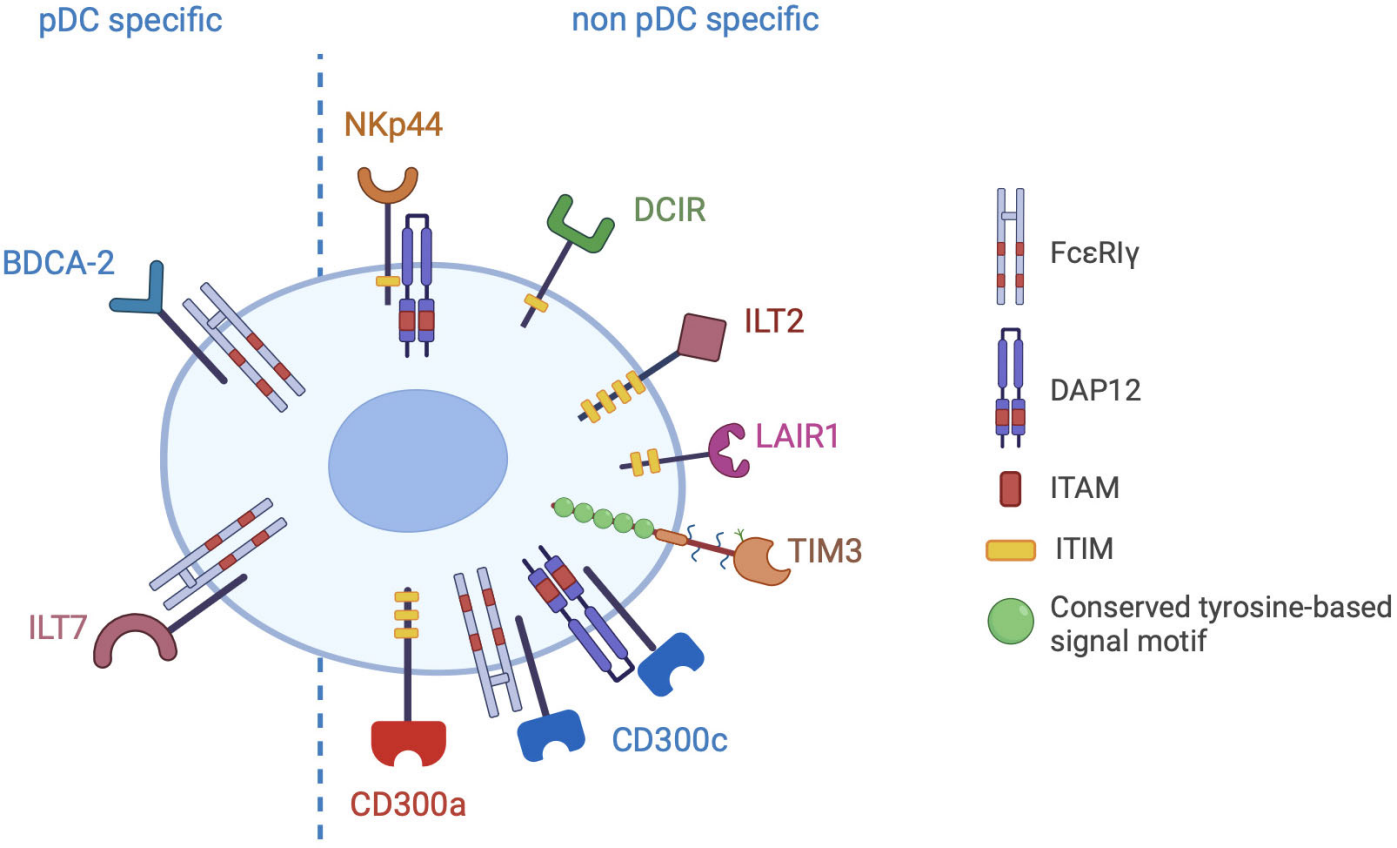
Inhibitory receptors of pDCs. The picture shows the inhibitory receptors (IRs) specifically expressed by pDCs (pDC specific) or shared with other cell types (non pDC specific). IRs signal through ITAM/ITIM motives present in the cytoplasmic domain of the receptor or in the associated adapter proteins (FcεRIγ or DAP12).

pDCs express a large variety of membrane receptors, either specifically expressed or shared with other immune and non-immune cells, conveying inhibitory signals that decrease the production of type I IFNs (**Figure 3**). The physiological significance of these receptors is preventing aberrant immune activation. Indeed, a deregulated and prolonged exposure to IFNs not only can increase the risk of autoimmunity, but can also interfere with haematopoiesis leading to lymphopenia (111, 112). Therefore, in homeostatic conditions, the engagement of IRs ensures a specific and brief IFN secretion and is crucial to maintain efficient immune responses while preventing immune-mediated tissue damage. However, IRs can be hijacked by pathogens or tumour cells, thus hindering pDC activation. Here, we will describe pDC-expressed IRs, emphasizing available evidence for their hijacking in cancer as well as novel blocking strategies aimed at rescuing the anticancer potential of pDCs.

### 4.1 BDCA-2

BDCA-2/CD303/CLEC4C is a human pDC-specific phenotypic marker (**Figure 3**), downregulated upon pDC maturation and TLR7/TLR9 triggering (113) and upregulated by IFN-α (37), and was the first receptor identified to negatively regulate the IFN response of pDCs (114). Lately, it was also shown to inhibit the TLR-mediated induction of TNF-related apoptosis-inducing ligand (TRAIL), thus impairing the capability of activated pDCs to kill TRAIL receptor-expressing neoplastic or infected cells (115).

BDCA-2 belongs to C-type lectin receptor (CLR) superfamily, as named after the Calcium-dependent binding of the first identified member. To date, CLRs are subdivided in 17 subgroups according to their structure, ligands and phylogeny (116). BDCA-2 belongs to the group II of CLRs that also includes, in humans, the closely related Dectin-2, dendritic cell immunoreceptor (DCIR, described further), DC immune-activating receptor (DCAR), and other members (117). These receptors are type II transmembrane proteins, with an extracellular C-terminal domain containing the carbohydrate recognition domain (CRD), and a short intracellular tail. In general, CLRs bind glycosylated molecules, a capability exploited by immune cells to recognize glyco-conjugated structures in non-self (pathogen-associated molecular patterns, or PAMPs), damaged-self (damage-associated molecular pattern, or DAMPs) and altered-self molecules (e.g. tumour-associated molecular patterns, or TAMPs) (118). Sequence analysis of the BDCA-2 CRD showed the presence of the tripeptide motif EPN (Glu-Pro-Asn), predicting the selective binding to the equatorial configuration of the hydroxyl groups at C3 and C4 of mannose, glucose, N-acetylglucosamine and fucose (117). Crystallographic analysis of the core domain of BDCA-2 CRD showed that its basic architecture is coherent with a typical CLR constituted by two α-helices and five β-strands (119). Differently from the CRD of other CLRs, a long loop region connecting α2-helix to β3-strand suggests the formation of a domain-swapped dimer, devoid of carbohydrate-binding ability, which may represent a regulatory mechanism that preserves BDCA-2 binding to galactosylated proteins in the Golgi apparatus before membrane exposure (119).

The nature and identity of BDCA-2 ligands is eagerly being sought after. Curiously, in contrast with the predicted mannose, N-acetyl glucosamine and glucose residues (120), a glycan array identified asialo-oligosaccharides with terminal galactose as BDCA-2 ligands (121). The binding ability for galactose–terminated glycans was subsequently ascribed to the interaction with a secondary site rather with the primary calcium-dependent binding site (122). Because serum glycoproteins display asialo-galactose residues, these were hypothesized to represent BDCA-2 ligands. Indeed, IgG, IgA, IgM but also α2-macroglobulin were demonstrated to bind BDCA-2, even if with low affinity (123). In the lack of any evidence of pDC activation following this binding, it was speculated that serum glycoproteins could compete with other ligands to maintain circulating pDCs in a quiescent state. In line with this, altered IgG galactosylation was described in autoimmune diseases characterized by pDC activation such as rheumatoid arthritis, primary Sjogren’s syndrome, psoriatic arthritis, and systemic lupus erythematosus (SLE) (124). Among PAMPs, different molecules from both DNA and RNA virus were shown to bind BDCA-2, possibly contributing to pDC exhaustion observed in chronic infections and, albeit for short term, during the acute phase of LCMV, HSV-1, VSV and MCMV infections (106). Recently, HBsAg, HIVgp120 and non-structural-1 (NS1) glycosylated protein from Zyka virus were shown to bind BDCA-2 and activate its downstream signaling pathways, leading to impaired type I IFN production upon TLR7 or TLR9 triggering (125–127). Tumors exploit modifications of cell surface carbohydrates to increase cell adhesion and migration, thus promoting invasiveness and metastasizing, but also to elude effective immune responses (128–130). For instance, carbohydrate changes of the carcino-embryonic antigen expressed by human colorectal cancer cells trigger the CLR DC-SIGN, which inhibits DC maturation and antitumor T cell activation by (131). Similarly, experiments exploiting a fluorescent tetramer encoding the BDCA-2 CRD, showed that BDCA-2 binds to tumor cells (including ovarian, colon, pancreatic carcinoma and breast adenocarcinoma) but not to non-tumor cells such as primary B and T cells (121). Cells expressing BDCA-2 ligands impaired the production of IFN-α following TLR9 stimulation, while ligand-negative cells did not, unless pre-treated with neuraminidase, which unmasks BDCA-2 binding sites (121). These findings suggest that tumor cells may modulate the expression of glycoproteins as a mechanism to inhibit human pDCs via BDCA-2 triggering.

**Figure 4:**
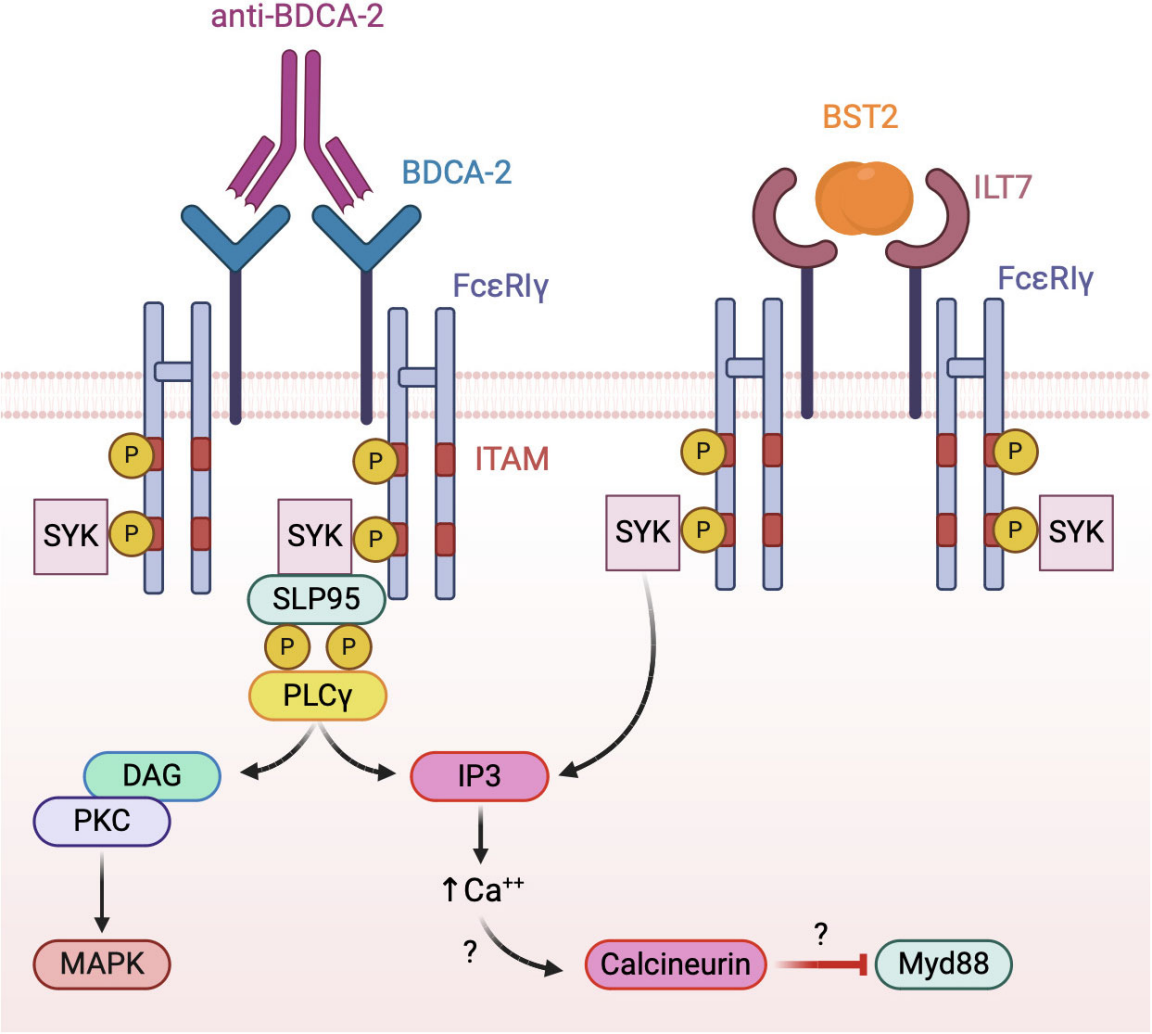
Model of BDCA-2 and ILT7 signaling. Both receptors associate with the ITAM-bearing adapter FcεRIγ chain. Receptor triggering activates a BCR-like signalosome leading to the inhibition of TLR signaling, possibly interfering with the TLR adapter MyD88.

To date, the only known function of BDCA-2 is the inhibition of TLR-dependent pDC activation. However, given the lack of well-defined biological BDCA-2 ligands, most studies investigating the mechanisms of BDCA-2 activation in pDCs were performed with crosslinking antibodies. As a result, the nature, the affinity and the kinetics of BDCA-2 triggering by natural ligands remains largely unknown and need further elucidation. Available results indicate that BDCA-2 signal transduction relies on the association with the common gamma chain of the Fcϵ receptor (FcϵRIγ), driving the assembly of a B cell receptor-like signalosome (132, 133) (**Figure 4**, left panel). Indeed, BDCA-2 triggering promotes the activation of the tyrosine kinase Syk that recruits the adaptor protein SLP65, leading, in turn, to phospholipase Cγ2 (PLCγ2) activation with the release of inositol 1,4,5-triphosphate and diacyl-glycerol. These second messengers are required for diverse membrane functionality including calcium flux. BDCA2 engagement has also been associated to AKT and MEK1/2-ERK activation (134, 135). To date, the pathway leading to BDCA-2 inhibition of TLR-dependent NF-kB activation remains partially elucidated (133), but PLCγ2 activation and calcium mobilization were suggested to impair the recruitment of MyD88 to TLRs through the activation of the serine phosphatase calcineurin (128, 136) (**Figure 4**, left panel).

Because of its specificity and inhibitory function, BDCA-2 is an attractive candidate for therapeutic strategies aimed at targeting pDCs or at modulating their activity. Bivalent binding by the F(ab)2 domain of anti-BDCA-2 antibodies is essential for BDCA-2 activation (137), while Fc region involvement seems to be dispensable. In fact, anti-BDCA-2 antibodies devoid of effector functions block the production of type I IFNs by TLR7/9-activated pDCs. However, the Fc region of a humanized monoclonal antibody against BDCA-2 appeared critical for inhibiting the production of type I IFN stimulated by immune complexes through internalization of CD32a (138). Regardless this difference, anti-BDCA-2 antibodies appeared as appealing tools for the treatment of SLE, where immune-complexes and type I IFNs play a pathogenetic role. A recent Phase II clinical trial involving patients with SLE demonstrated that Litifilimab (a humanized antibody against BDCA-2) could reduce cutaneous and joint involvement (LILAC ClinicalTrials.gov number NCT02847598) (139, 140). Another strategy to target BDCA-2 was the generation of a chimeric anti-BDCA-2 antibody (ch122A2), characterized by low fucose contents in order to increase its affinity for CD16/FcγRIIIa to activate the antibody-dependent cell-mediated cytotoxicity. In preclinical settings, ch122A2 induced an efficient and fast depletion of blood pDC in humanized mice (141). A proposed clinical application of this antibody is primarily the treatment of patients with pDC malignancy like BPDCN or pDC-AML; however, other hematological and solid cancers where pDC infiltration associates with a poor prognosis may be potential therapeutic targets.

### 4.2 ILT7

ILT7/LILRA4/CD85g is the only other pDC-specific IR in humans (142–144) (**Figure 3**). Similarly to BDCA-2, its expression is upregulated by IFN-α (37) and downregulated when pDCs are activated by TLR agonists or treated with the survival cytokine IL-3 (142), possibly as a result of reduced transcriptional expression by activated pDCs (145).

The ILT (also known leukocyte Ig-like receptor-LILR- or monocyte Ig-like receptor-MIR-) gene family is composed of 11 transmembrane proteins characterized by two or four extracellular C2-type Immunoglobulin-like domains. ILTs are expressed by various population of antigen presenting cells in humans and primates but not in rodents (144, 146). Sequence analysis revealed the existence of separate subgroups of ILTs: one characterised by a long intracellular tail bearing immunoreceptor tyrosine-based inhibitory motifs (ITIMs), one devoid of any transmembrane domain, and one with short cytoplasmic tails without ITIMs but characterized by the presence of a charged residue in the transmembrane domain, which allows the association with signalling adapter molecules that possess ITAMs.

ILT7, characterized by four extracellular immunoglobulin domains, belongs to the latter subgroup, displaying a positively charged arginine residue at position 449 within the predicted transmembrane segment, through which it associates with FcϵRIγ, the same ITAM-bearing adapter used by BDCA-2 (142). Similar to BDCA-2, both Src family kinases and Syk are rapidly phosphorylated after ILT7 crosslinking in human primary pDCs indicating the onset of ITAM signaling together with prominent intracellular calcium mobilization (142) (**Figure 4**, right panel).

In the search for ligands, ILT7 reporter cells were found to be activated in the presence of human breast carcinoma cells and melanoma cell lines but not by common laboratory mammalian cell lines (143). Bone marrow stromal cell antigen 2 (BST2; CD317) was identified as the ILT7 ligand capable of inducing changes similar to those observed upon cross-linking by anti-ILT7 antibodies (143). When ILT7 was cross-linked by either anti–ILT7 antibodies or recombinant BST2 protein, pDCs stimulated by the TLR9 ligand CpG oligonucleotide and TLR7 ligand influenza virus produced less IFN-α and TNF-α. By contrast, the expression of the costimulatory molecules CD80 and CD86 was not affected (142, 143). Given that BST2 is robustly induced by IFNs and inflammatory cytokines, its interaction with ILT7 was identified as a negative feedback mechanism to prevent prolonged IFN production after viral infection (147). In accordance with original experiments showing constitutive expression of BST2 by cancer cells (143), more recent observations confirmed BST2 overexpression in myelomas, lung cancer, breast cancer, colorectal cancer, and pancreatic cancer (148), which could represent a strong predictor of tumor size, aggressiveness, and poor patient survival (149, 150). *In vitro*, BST2 expression by human breast cancer and melanoma cell lines could suppress the production of type I IFNs via ILT7 (143). Of interest, ILT7 was recently found to be modulated in tumor-infiltrating pDC of melanoma patients (151). These pieces of evidence strongly suggest that the interaction of BST2 with ILT7 may contribute to tumor immune suppression and pDC–tumor crosstalk (144).

### 4.3 Non pDC-specific IRs

Human pDCs express several other membrane receptors conveying inhibitory signals that, unlike BDCA-2 and ILT7, are also expressed by other immune or non-immune cells.

Two of these receptors, NKp44 and DCIR, are CLRs (group V and II, respectively) like BCDA-2, but both express intracellular ITIMs (**Figure 3**), which are normally involved in the inhibition of kinase-mediated signals by recruiting tyrosine phosphatases like Src homology region 2 domain-containing phosphatase (SHP)-1 or -2. The ITIM sequence of NKp44 was originally shown to be non-functional in the attenuation of NK-like cells activation (152), thus classifying it as a NK cell-triggering receptor. However, its ligation by the ligand proliferating cell nuclear antigen (PCNA) was later found to deliver ITIM-dependent inhibitory signals into NK cells (153). Thus, in NK cells NKp44 works as a dual function receptor, possibly depending on the streghth of its engagement as described for many CLRs (154). PCNA overexpression is a hallmark of cancer virulence and promotes cancer survival via several mechanisms, including immune evasion through inhibition of NKp44-mediated NK cell attack. Consistent with this view, downregulation of endogenous PCNA in pancreas, prostate, breast and brain tumor cell lines by a siRNA approach and the blockade of NKp44-PCNA interaction in triple negative breast cancer cells by a monoclonal antibody increased NK cytotoxicity and tumor killing (155). NKp44 is also constitutively expressed by a small subset of tonsil pDCs and can be induced in blood pDCs by IL-3 stimulation. In pDCs, NKp44 crosslinking by a specific antibody inhibited the production of IFN-α in response to TLR9 agonists via the association with the ITAM-bearing adaptor protein DAP12 (156). In PCNA+ human melanoma, infiltrating pDCs showed increased NKp44 levels, which correlated with a low activation level, suggesting that the interaction of NKp44 with PCNA expressed by melanoma cells could contribute to pDC dysfunctions typically observed in melanoma patients. In addition, melanoma patients displaying higher frequencies of NKp44+ pDCs in their blood were more likely to have worse clinical outcome (151). However, it was recently demonstrated that NKp44 engagement by dimers of platelet derived growth factor (PDGF-DD), another physiologic ligand, enhanced the secretion of IFN-α induced by a TLR9 ligand (157) suggesting that NKp44 possibly works as a dual function receptor also in pDCs and that its inhibitory role needs to be re-assessed in each specific tumor context.

DCIR (also known as CLEC4A) is expressed on a variety of immune cells such as cDCs, B cells and monocytes/macrophages in addition to pDCs. Due to the presence of an intracellular ITIM domain (**Figure 3**), it is generally regarded as an IR (158), although, like other CLRs (including the above mentioned NKp44) it can deliver activatory signals in certain cell types and conditions (154). In human pDCs, DCIR crosslinking inhibited TLR9-induced IFN production (53, 159). In respect with BDCA-2, pDC inhibition by DCIR was less effective and TLR9-specific since it could not be observed when pDCs were stimulated with TLR7 ligands (53). Very recently, asialo-biantennary N-glycans were shown to represent a DCIR functional ligand, capable to regulate DC functions in both humans and mice (160). However, DCIR was previously shown to interact with several ligands of both pathogenic and endogenous origin (129). In pDCs, DCIR binding by HCV glycoprotein E2 inhibited the production of type I IFNs by HCV particles through a rapid AKT and ERK1/2 phosphorylation (134). The recognition of self-glycans by DCIR prevented autoimmunity in murine models of rheumatoid arthritis (159). In cancers, DCIR could recognize aberrant glycosylation in prostatic, gastric and colon cancer human cell lines (129). In a mouse model of inflammation-induced colorectal cancer, the administration of antibodies blocking the interaction of DCIR with asialo-biantennary N-glycans reduced tumor incidence by reverting the DCIR-dependent blockade of alarmin recognition by TLRs, suggesting a crucial role for DCIR in the maintenance of the intestinal immune system functionality and that DCIR may represent a promising target for the treatment of colitis and colon cancers (161). Additionally, skin delivery of DCIR small hairpin RNA delayed tumor growth in mouse models of bladder and lung tumor by enhancing T cell mediated immunity and also potentiated the anti-tumor effects of a DNA vaccine (162).

ILT2, unlike ILT7, bears four intracellular ITIM motifs (**Figure 3**) and is broadly expressed on blood pDCs, monocytes, B cells, cDCs, NK cell subsets and T cells (163). ILT2 engagement significantly suppresses the ability of DC subsets, including pDCs, to produce cytokines, upregulate costimulatory molecules, and stimulate T-cell proliferation (164–166). In humans, whole blood stimulation with TLR4 and TLR7 agonists increased membrane expression of ILT2 in pDCs and, consistent with its immunosuppressive role, IL-10 treatment during TLR stimulation further increased ILT2 expression (167). ILT2 recognized pathogens as well as endogeous ligands (165, 166), particularly non-classical MHC class I molecules (163). Among them, HLA-G expression has been described in several tumor types, where it contributed to malignant progression by contrasting immune surveillance via the interaction with ILT2 and ILT4 (168). In accordance, anti-HLA strategies were recently proposed as novel immune checkpoint inhibition approaches in solid cancers (169). In chronic lymphocytic leukemia, ILT2 expression was significantly decreased on leukemic cells and increased on NK cells, particularly in patients with advanced disease and with poor prognostic features. ILT2 suppressed NK cell activity, which could be restored by ILT2 blockade: in combination with the immunomodulatory drug lenalidomide, ILT2 blockade potentiated the elimination of human leukemic cells (170). Disruption of ILT2 activation with blocking monoclonal antibodies increased NK cell-mediated IFN-γ production and cytotoxicity against human glioblastoma cell lines, partially reverting the immunosuppression linked to this malignancy. In addition, co-treatment with temozolomide strengthened the antitumor capacity of immune cells treated with anti-ILT2 (171). Also, Fc-silent antibodies against ILT2 significantly enhanced antibody-dependent phagocytosis of lymphoma cell lines when combined with both rituximab and blockade of CD47 (172). These findings suggest that the blocking of ILT2 may be an interesting strategy to improve tumor immunotherapy.

Leukocyte-Associated Ig-like Receptor-1 (LAIR1) is an ITIM-bearing immune-IR expressed by the majority of immune cells, including T cells, B cells, NK cells, monocyte/macrophages, neutrophils, pDCs, as well as by tumor cells (173). Crosslinking of LAIR1 in human pDCs inhibited TLR-dependent type I IFN production, displaying a coordinated regulatory function with NKp44 (156, 174). Four different types of ligands are described, including components of the complement system and collagens, suggesting a potential immune-regulatory function of the extracellular matrix (175, 176). In a retrospective study, LAIR1 expression was found to associate to poor prognosis in invasive breast carcinoma (177), but also to resistance to PD1/PD-L1 inhibition in patients (178). Of interest, LAIR1 blockade by antagonist antibodies inhibited tumor development in a humanized mouse model by affecting, among others, the recruitment of pro-tumorigenic pDCs (179). In addition to blocking antibody, LAIR1-inhibitory signaling can be blocked also by taking advantage of LAIR2, a natural agonist (180) as proposed by a work using a dimeric LAIR2 Fc fusion protein to target collagens in tumors and reverse immune suppression (181). The potential of LAIR1 blockade in cancer immunotherapy is currently emerging (179, 182). However, since LAIR1 is widely expressed also by tumor cells, where it may induce either proliferation or inhibition depending on the tumor type (173), its therapeutic exploitation needs to be carefully tailored to each specific cancer microenvironment.

T cell immunoglobulin and mucin domain-containing protein 3 (TIM3) is a member of the TIM family of immunoregulatory proteins expressed by pDCs, T cells, regulatory T cells, NK cells, and myeloid cells. TIM3 lacks intracellular inhibitory signaling motifs and the precise intracellular signalling mechanism remains poorly elucidated (183). Different mechanisms were proposed for TIM-3-induced suppression of IFN production in pDCs activated by nucleic acids. Chiba and colleagues highlighted the ability of TIM3 to bind and sequester HMGB1 away from TLR, thus avoiding the sensing of tumour-derived nucleic acids bound to HMGB1 itself. In contrast, Schwartz and colleagues suggested that TIM3 could act by recruiting IRF7 into acidic lysosomes, thus promoting the degradation of proteins important for IFN-α production (184, 185). Tumour cells can exert immunosuppression by expressing TIM3 ligands such as galectin-9 and CEACAM-1 (186, 187). Increased serum levels of galectin-9 was found in cancer patients and predicted poor response to treatment in high grade serous ovarian carcinoma and in adult leukemia patients (188). Consistent with a suppressive function in the tumour microenvironment, TIM3 was found upregulated in lung tumor-infiltrating pDCs (167, 184). One group showed that galectin-9 could block TLR-induced pDC activation *in vitro* and in a murine model also via the engagement of CD44 (189), a widely-expressed adhesion receptor involved in cancer metastasizing and regulation of T cell responses (190). In human pDCs, CD44 engagement by galectin-9 impaired mTOR-dependent TLR activation (189).

Finally, also the triggering of the CD300a/c glycoproteins by crosslinking antibodies was shown to decrease type I IFN and TNF-α secretion by human pDCs stimulated with TLR7 and TLR9 ligands (191). CD300 are a group of type I transmembrane receptors belonging to the B7 family with a single IgV-like extracellular domain containing 2 disulfide bonds (192). While CD300a contains several ITIMS in its long intracytoplasmic domain, CD300c associates with the ITAM-bearing adapters DAP12 and/or FcϵRIγ via a transmembrane glutamic acid residue (192) (**Figure 3**). Both receptors are expressed by virtually all leukocytes and possibly recognize lipids that are exposed on the outer leaflet of the plasma membrane of dead and activated cell. CD300 receptors are also highly expressed by human cancer cells, especially in acute myeloid leukemia (193). To date, their therapeutic potential in cancer immunotherapy remains to be elucidated.

## 5 Therapeutic strategies to restore the antitumor potential of pDCs

Although being a minor population both in the circulation and in the tumor microenvironment, evidence described in Section 3 indicates pDCs as interesting targets for anticancer immunotherapy. Several therapeutic protocols have been developed to this end, mostly aiming at reverting the distinctive feature of immunosuppressive TA-pDCs, i.e. the impaired secretion of type I IFNs.

### 5.1 TLR stimulation

The most used approach to stimulate pDC production of type I IFNs is TLR7 and TLR9 stimulation, either individually or in combination (194). Indeed, TLR9 engagement by intralesional administration of CpG ODN nanorings gave promising results in a thymoma mouse model, where the increased production of IFN-α by pDCs associated to reduced tumor size and volume (195, 196). In a melanoma mouse model, CpG-activated pDCs were indispensable to induce CD8+ T cell antitumor response through cDC activation (197, 198). Single-stranded RNAs delivered by the positively charged protein protamine promoted T cell proliferation, demonstrating that protamine–RNA complexes can be used to stimulate human DC subsets ex vivo for future immunotherapeutic settings (199). The potent synthetic TLR7 agonist imiquimod, approved for the treatment of basal cell carcinoma (200), was shown to increase the infiltration of activated pDCs into melanoma lesions and its combination with monobenzone led to metastases regression in phase II clinical trial in late-stage melanoma patients (201). Vidutolimod (202), a virus like particle containing a TLR9 agonist known as G10, enhanced IFN-α production by pDCs showing high therapeutic efficacy when administered alone or in combination with an anti-PD-1 therapy in patients with melanoma (203, 204). The combined stimulation with TLR agonists and FLT3L, a growth factor of both cDCs and pDCs, enhanced cDC antigen presentation and T cell immunity in mouse models of melanoma (205) and glioma (206). In line with these observations, the combined administration of imiquimod, FLT3L and a peptide-based vaccine not only increased the number of peptide-specific CD8+ T cells but also prompted the mobilization of cDCs and pDCs in melanoma patients (207). However, selective delivery of TLR7/9 agonists to pDCs *in vivo* still needs improving.

### 5.2 IR targeting

IR blockade may represent an alternative pDC-boosting strategy, especially in tumors characterized by high levels of inhibitory ligands or when TLRs are desensitized by continuous stimulation with exogenous or endogenous ligands.

**Figure 5:**
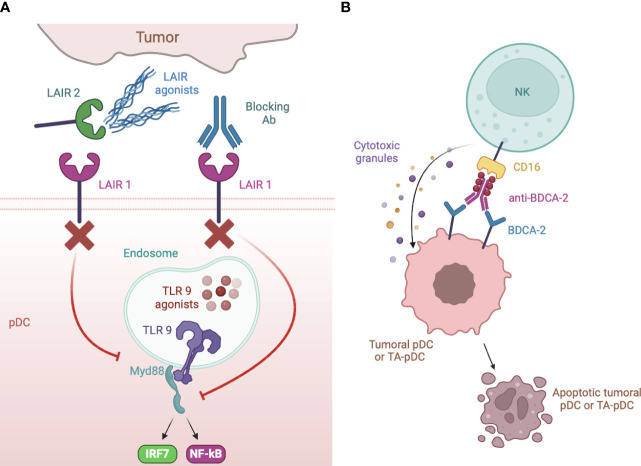
Strategies of IR targeting. **(A)** LAIR1 can be blocked to rescue type I IFN production by specific blocking antibodies or by a dimeric LAIR2 Fc fusion protein that sequesters ligands to membrane LAIR1. Blocking antibody were also developed against DCIR, ILT2, NKp44 and TIM3. **(B)** anti-BDCA-2 antibodies manipulated to increase their affinity for CD16 induce apoptotic cell death of neoplastic but also, possibly, TA- pDCs by NK cell activation.

Many cancer types express *de novo*, or overexpress, IR ligands which often represent a strong predictor of poor outcome and metastasis, as observed for BST2 (148), PCNA (208), HLA-G (168), galectin-9 (186), CEACAM-1 (186, 187) and modifications of cell surface carbohydrates capable to trigger CLRs (128–130). It is conceivable that these ligands abundantly expressed in cancers contribute to pDC exhaustion by engaging IRs. Indeed, ligand-expressing tumor cells were found to block pDC activation by engaging BDCA-2 (121), ILT7 (143), TIM3 (167, 184) or CD44 (189). In this setting, therapeutic strategies preventing receptor engagement or signaling would revert TA-pDC blockade (**Figure 1**). Among such strategies, blocking antibodies were developed against LAIR1 (179), DCIR (161), ILT2 (170, 171), NKp44 (155) and TIM3 (2) (**Figure 5A**). As alternative strategies, LAIR1 inhibition was also achieved via an Fc fusion protein of LAIR2, a natural agonist capable of sequestering the ligands (181) (**Figure 5A**), while DCIR expression was decreased by skin delivery of specific small hairpin RNA (162) (**Figure 5A**). As a matter of facts, TIM3 and, to some extent, ILT2 are already promising emerging targets for checkpoint blockade (2). Also, DCIR blockade was recently shown to reduce the incidence of experimental inflammation-induced colon carcinoma (161) and the potential of LAIR1 blockade in cancer immunotherapy is rapidly emerging (179, 182). However, these IRs are expressed by different immune and tumor cells and in most studies the neat contribution of pDC rescue in the elicited antitumor response is difficult to deduce or not addressed at all. Unfortunately, blocking antibodies for pDC-specific IRs, namely BDCA-2 and ILT7, are currently unavailable. However, BDCA-2 signaling could be blocked *in vitro* by saturating ligand-expressing cells with a tetramer encoding the BDCA-2 CRD or by treating them with β-(1–4)-galactosidase which removes terminal galactose that are crucial for BDCA-2 triggering (121, 128–130). In addition, an anti-BDCA-2 monovalent Fab was unable to activate BDCA-2 and to inhibit type I IFN production (137) suggesting that low avidity, monovalent antibodies could be exploited as therapeutic strategy to block BDCA-2 activation in tumors. Finally, a BDCA-2-binding antibody engineered to favor the activation of cytotoxicity efficiently depleted blood pDCs in humanized mice (141), possibly representing a primary tool for the treatment of pDC malignancies but also of other cancers where the presence of pDCs associates with a poor prognosis (**Figure 5B**).

## 6 Conclusions and future directions

This review summarizes the current knowledge on the pathophysiology of IRs expressed by pDCs, with a particular emphasis on their hijacking in cancer contexts, where pDC functions are generally reduced or abrogated. This evidence provides a proof of concept that the regulation of IFN secretion by pDCs may be regarded as an “innate checkpoint” which, similar to the “classical” adaptive immune checkpoint PD1 expressed in CD8+ T cells, restrains autoimmunity and immunopathology but may favor chronic infections and tumors. Accordingly, IRs may represent potential targets for innate and adaptive combined cancer immunotherapy to unleash T-cell–mediated tumor killing.

To date, however, the therapeutical exploitation of IR blockade to revert pDC exhaustion is hampered by several unanswered questions. First, the biology of pDCs in tumors is incompletely understood. For example, in the context of hepatic ischemia-reperfusion injury following surgical removal of hepatocellular carcinoma, tolerogenic pDCs associated to a better prognosis since type I IFNs crucially contributed to early tumor recurrence (209). Furthermore, OX40+ pDCs were found indispensable to activate cDCs and stimulate an efficient antitumor CD8+ T cells response in a mouse model of squamous carcinoma, which growth accelerated upon pDC depletion (210). pDC exhaustion may also be tumor- and even stage-specific, as recently shown in colon cancer where the presence of activated pDCs, as assessed by nuclear localization of IRF7, associated with increased patient survival (211). Type I IFNs themselves do play a dual role in cancer immunity, being protective in the early phases, while increasing the expression of PD1 and PD-L1 upon prolonged exposures (212). Thus, timing and duration of pDC activation may represent critical parameters in antitumor immune responses, requiring to be thoroughly understood and tightly regulated depending on the specific tumor context (69, 70). Also tumor-specific mechanisms of pDC suppression are incompletely known, not only in terms of IR ligand expression, but also concerning the distribution of IRs on pDC subsets and the possibility of their simultaneous engagement: *in vitro*, the engagement of one single IR is sufficient to block pDC activation, but no studies so far addressed the result of multiple engagement nor any possible hierarchical relationship among IRs. Finally, the expression patterns of “classical” immune checkpoint receptors (and ligands) on pDC subsets, poorly known to date, could affect the results of combined checkpoint inhibitor therapies.

We also mentioned that the avidity of IR engagement may influence to the final response of pDC (213). This is particularly relevant when IR inhibitory role is assessed by using crosslinking antibodies, that generally bind with high avidity, for example in the lack of specific ligands, but may hold true also for natural ligand endowed with different affinity. In the case of NKp44, PCNA overexpression by tumors sustained immune evasion through NKp44-mediated inhibition of both NK cells and pDCs (151), in accordance with the inhibitory role described by using crosslinking antibodies (155). In striking contrast, NKp44 engagement by PDGF-DD increased the production of type I IFNs by human pDCs activated with a TLR9 agonist (but, notably, not with a TLR7 agonist) (157). Thus, the role of IRs may differ in specific cancer context as well as their potential as therapeutic targets. However, such IR feature may also be exploited for the design of therapeutic tools, as demonstrated by the different activity of monovalent Fab fragment or cross-linking bivalent anti-BDCA-2 antibodies (137)

Last but not least, despite some IRs such as TIM3 and ILT2 are recognized targets for checkpoint blockade (2), they are widely expressed on immune and even tumor cells and the neat contribution of pDC exhaustion in tumor growth and the actual therapeutic significance of pDC rescue via IR blockade remains difficult to assess. By contrast, the anticancer potential of BDCA-2 and ILT7 blockade received little attention so far, partly depending on the lack of specific reagents. These IRs definitely deserve attention as targets in pathological conditions where pDC-specific modulation is required or pDC depletion can be advantageous.

In conclusion, despite encouraging evidence, more work is required to fully unravel the effects of IR engagement on pDC functions in specific tumor microenvironments and to uncover the beneficial role of therapeutic blockade of pDC-specific IRs in future immunotherapeutic strategies.

## Author contributions

LT: Conceptualization, Writing – original draft. ML: Writing – original draft. GZ: Visualization, Writing – original draft. VS: Writing – review & editing. TS: Writing – review & editing. SS: Conceptualization, Funding acquisition, Supervision, Writing – review & editing. ADP: Conceptualization, Supervision, Writing – review & editing. DB: Conceptualization, Funding acquisition, Supervision, Writing – review & editing.
